# G protein regulation by RGS proteins in the pathophysiology of dilated cardiomyopathy

**DOI:** 10.1152/ajpheart.00653.2024

**Published:** 2025-01-07

**Authors:** Yadhira E. Garcia, Benita Sjögren, Patrick Osei-Owusu

**Affiliations:** 1 Department of Pharmaceutical Sciences, University of California, Irvine, California, United States; 2 Department of Biological Chemistry, University of California, Irvine, California, United States; 3 Department of Physiology and Biophysics, Case Western Reserve University School of Medicine, Cleveland, Ohio, United States

**Keywords:** dilated cardiomyopathy, G protein signaling, pathological mechanisms, RGS proteins, vascular dysfunction

## Abstract

Regulators of G protein signaling (RGS) proteins fine-tune signaling via heterotrimeric G proteins to maintain physiologic homeostasis in various organ systems of the human body including the brain, kidney, heart, and vasculature. Impaired regulation of G protein signaling by RGS proteins is implicated in the pathogenesis of several human diseases including various forms of cardiomyopathy such as hypertrophic cardiomyopathy and dilated cardiomyopathy (DCM). Both genetic and nongenetic changes that impinge on G protein signaling in cardiomyocytes are implicated in the etiology of DCM, and there is accumulating evidence that such genetic and nongenetic changes affecting G protein signaling in cell types other than cardiomyocytes could serve as a DCM trigger in humans. This review discusses and highlights mammalian RGS proteins and their roles in cardiac physiology and disease, with a specific focus on the current understanding of the etiology of DCM and the pathogenic roles of RGS proteins that are prominently expressed in the cardiovascular system. Growing evidence suggests that defects in G protein regulation by RGS proteins in the cardiovascular system likely contribute to cardiomyocyte structural damage and decreased contractile function that hallmark DCM. Further studies that enhance the understanding of the dynamics of G protein regulation by RGS proteins in several cell types in the myocardium and the vasculature are critical to gaining more insight into the etiology of DCM and heart failure, and to the identification of novel therapeutic targets.

## INTRODUCTION

Cardiomyopathies are disorders of the heart and comprise a variety of structural and functional abnormalities, mainly of cardiomyocytes. Cardiomyopathies may be a consequence of overt coronary artery disease or acute myocardial infarction leading to cardiomyocyte death and subsequent ventricular decompensation and chamber dilation (1–3). Nonischemic cardiomyopathies, including hypertrophic cardiomyopathy, arrhythmogenic right ventricular cardiomyopathy, left ventricular noncompaction, and dilated cardiomyopathy (DCM) are characterized by left ventricular chamber dilatation and contractile dysfunction without other hallmark cardiovascular disorders such as hypertension, severe coronary heart disease, valvular disease, or congenital heart disease (1, 3). Recent advances in the understanding of disease etiology have led to new classifications of DCM as primary or secondary cardiomyopathy. Accordingly, DCM with underlying genetic mutations (whether spontaneous or familial) in the absence or presence of nongenetic factors but restricted to the heart are classified as primary. On the other hand, secondary DCM is that associated with or is a component of a systemic disease such as Gaucher disease, Fabry disease, sarcoidosis, amyloidosis, systemic lupus erythematosus, or a complication resulting from cancer therapy (1). Considering just the reported or appropriately documented cases in the general population, the annual incidence of DCM is roughly 5–7 cases per 100,000 people, while prevalence is estimated to be between 1:200 and 1:400 (2–4), which is only slightly less than the restrictive and the more common type of cardiomyopathy, hypertrophic cardiomyopathy (HCM) (1, 5). Currently, DCM remains one of the most common causes of heart failure (5, 6). The etiology of DCM is heterogeneous and can be hereditary (genetic), acquired (nongenetic), or mixed, especially in cases where there is a substantial overlap between the two main causes (1). DCM may be further classified as idiopathic dilated cardiomyopathy (IDCM); these are cases with unknown or ill-defined genetic and nongenetic factors (1, 7). Because several IDCM cases are also associated with other noncardiovascular diseases or disorders, such as alcoholism or cancer therapy, whether such IDCM is truly primary or secondary DCM is unclear. In any event, the nongenetic causes or factors underlying DCM may involve subtle changes in myocardial perfusion leading to reactive inflammation with exacerbation of cardiomyocyte damage or death, and the development of the hallmark myocardial fibrosis (3, 8). Although overt coronary artery disease is atypical of DCM, subtle changes in the structure and function of the vasculature may lead to chronic coronary hypoperfusion sufficient to trigger local inflammatory response in the myocardium. However, whether aberrant changes in vascular structure and function are a cause or a primary contributor to DCM pathogenesis is not clear and relatively less explored.

Accumulating evidence suggests that multiple cellular signaling pathways implicated in DCM pathogenesis involve certain anomalies in the regulation of signal transduction pathways activated by G protein-coupled receptors (GPCRs), including signaling via guanine nucleotide-binding (G) proteins. This review provides an overview of the molecular mechanisms underlying DCM, with a focus on the current understanding of changes in G protein signaling regulation by regulator of G protein signaling (RGS) proteins and related regulatory proteins in cardiomyocytes, vascular cells, and fibroblasts, as relates to DCM pathogenesis and progression to heart failure.

### RGS Proteins and Cardiac (Dys)Function

Signaling via heterotrimeric (Gα, Gβ, Gγ) G proteins in the myocardium has been studied extensively due, partly, to the multifaceted role of primary activators—GPCRs—in normal cardiac physiology and in disease pathogenesis, and also due to the broad expression of downstream signaling effectors and regulators in the cardiovascular and other organ systems. Among the key proteins that regulate G protein signaling are RGS proteins, a family of GTPase activating proteins (GAPs) broadly expressed in the myocardium. The GAP function of RGS proteins is conferred by the presence of a conserved Gα-binding domain (RGS domain) that defines all RGS proteins. RGS proteins mostly influence cardiac G protein activity via their characteristic RGS domain that functions as a GAP to accelerate the hydrolysis of GTP bound to the Gα subunit and render that subunit inactive (9). Inactivated, GDP-bound Gα quickly binds to Gβγ obligate dimer to reconstitute the inactive, GDP-bound heterotrimeric G protein ready to be activated by a nonreceptor activator or a cognate GPCR in an active conformation (10). Thus, by their action, RGS proteins regulate both the kinetics and amplitude of signaling downstream of heterotrimeric G proteins. To date, roughly 30 mammalian RGS proteins have been discovered (11, 12). Profiling of cardiac expression identified mRNA transcripts of all mammalian RGS proteins discovered to date in mammalian hearts, including mouse, rat, and human hearts (11).

RGS proteins are classified into four main families (R4, R7, R12, and RZ) based on sequence homology, the absence or presence of additional domains besides the conserved RGS domain, and selectivity for one or more of the four classes of Gα subunit (Gα_s_, Gα_i/o_, Gα_q/11_, and Gα_12/13_). The R4 family includes RGS1–5, 8, 13, 16, 18, and 21, the R7 family includes RGS6, 7, 9, and 11, the R12 family includes RGS10, 12, and 14, and the RZ family includes RGS17, 19, and 20 (13, 14). Of the RGS proteins covered in this review, RGS2 has selective GAP activity toward Gα_q/11_, whereas RGS3, 4, and 5 have similar GAP activity toward both Gα_q/11_ and Gα_i/o_, and RGS6, 7, 10, and 12 have GAP activity toward G_i/o_ (15). Although all RGS proteins display GAP activity toward Gα subunits, many of them also have functional effects unrelated to G proteins. In the majority of cases, the non-G protein effects are mediated by functional domains other than the RGS domain; however, the absence of additional functional domains does not necessarily exclude non-GAP functions of RGS proteins. For example, RGS2, which only has the well characterized RGS functional domain, has been shown to inhibit certain adenylyl cyclase (AC) isoforms, as well as inhibit total protein synthesis, both through interactions involving its amino (N)-terminal domain (16, 17).

Studies of cardiac physiology and disease have identified key roles of multiple RGS proteins in maintaining physiological homeostasis and in disease pathogenesis (Table 1). For instance, RGS3 is highly expressed in atria and has been found to blunt maladaptive cardiac hypertrophy when overexpressed. In addition, RGS3 overexpression in mouse hearts was found to improve cardiac function by inhibiting mitogen-activated protein kinases (MAPK)/extracellular signal-regulated kinase (ERK) signaling (39). RGS6, belonging to the R7 family of RGS proteins that regulate G_i/o_ signaling, was also found to negatively regulate cardiac vagal activity, and mice with a truncated variant of RGS6, and lacking the RGS domain, were found to have severe bradycardia due mostly to the loss of RGS6 GAP activity toward G_i/o_ (40). The GAP activity of RGS6 was also found to be crucial to the regulation of potassium currents, specifically, G-protein inwardly rectifying potassium (GIRK) channels, in cardiomyocytes. Moreover, cardiomyocytes expressing the “RGS-domainless” RGS6 variant were also found to have prolonged activation and slow desensitization of GIRK channels (40).

A few studies in recent decades have shed more light on the involvement of RGS proteins in the pathogenesis of various cardiomyopathies that progress to heart failure. Interestingly, and relevant to DCM pathogenesis, *Rgs6*^−/−^ mice were protected against anthracycline (doxorubicin)-induced DCM leading to heart failure (41). Based on the data showing that the absence of RGS6 decreased doxorubicin-induced myocardial cell apoptosis, the authors of that study concluded that RGS6 promotes doxorubicin-induced cardiomyopathy (41). Contrary to that conclusion, RGS6 was found to confer cardio-protection against ischemic injury, whereas Rgs6 deletion potentiated ischemic heart injury (42). Whether the cardioprotective effect of RGS6 in DCM and heart failure is inherent in the RGS domain that executes GAP activity, or in other domains of the protein, remains to be elucidated. Another RGS protein implicated in DCM pathogenesis is RGS12 of the R12 family. Like the other members of the R12 family, RGS12 modulates inhibitory signaling via Gα_i/o_ and thereby upregulates AC activity and signaling mediated by 3’,5’-cyclic adenosine monophosphate (cAMP) (9). RGS12 also acts as a GAP toward Gα_q/11_ class G proteins to reduce inositol 1,4,5-trisphosphate (IP_3_)-mediated Ca^2+^ release from internal stores, thereby promoting myocyte relaxation (9). A previous study involving human heart samples reported that patients with DCM had altered levels of RGS12 expression in the myocardium, and RGS12 protein levels were elevated in failing hearts from patients with DCM and HCM (38). Consistent with this finding, overexpression of RGS12 in mice led to increased cardiomyocyte cross-sectional area and subsequent decompensation to ventricular dilation and decreased cardiac function characteristic of heart failure from DCM (38). Moreover, marked elevation of RGS12 protein expression was found to associate with pressure-overload cardiac hypertrophy, whereas deletion of *Rgs12* was found to decrease cardiomyocyte cross-sectional area, with decreased mRNA levels of common biomarkers of maladaptive cardiac hypertrophy in mice (38). The putative mechanism underlying DCM associated with *Rgs12* appears to be alterations in MAPK/ERK1/2 signaling, based on the observed decreases in ERK1/2 phosphorylation in hearts from *Rgs12*^−/−^ mice (38).

Accumulating evidence continue to implicate multiple members of the R4 family of RGS proteins in various cardiomyopathies, and this may be related to their high expression, relative to other RGS families, throughout the myocardium. For instance, the expression of RGS4, which is notably prominent in the sinoatrial and atrioventricular nodal regions, was found to be markedly increased in myocardial samples from failing hearts of patients with DCM and ischemic cardiomyopathy (43). Augmented RGS4 expression was also found to be associated with decreased Gα_q_ signaling and negative modulation of GPCR-stimulated cardiomyocyte growth, contractility, and hypertrophy (43). Consistent with these observations, a study by Rogers et al. (44) found that RGS4 overexpression in mice ameliorated pressure-overload cardiac hypertrophy, with antihypertensive effects, thus leading to the hypothesis that the upregulation of RGS4 in the failing myocardium is part of a compensatory mechanism to protect the heart from inappropriate activation of Gα_q/11_ and downstream signaling leading to maladaptive remodeling.

**Figure 1.**
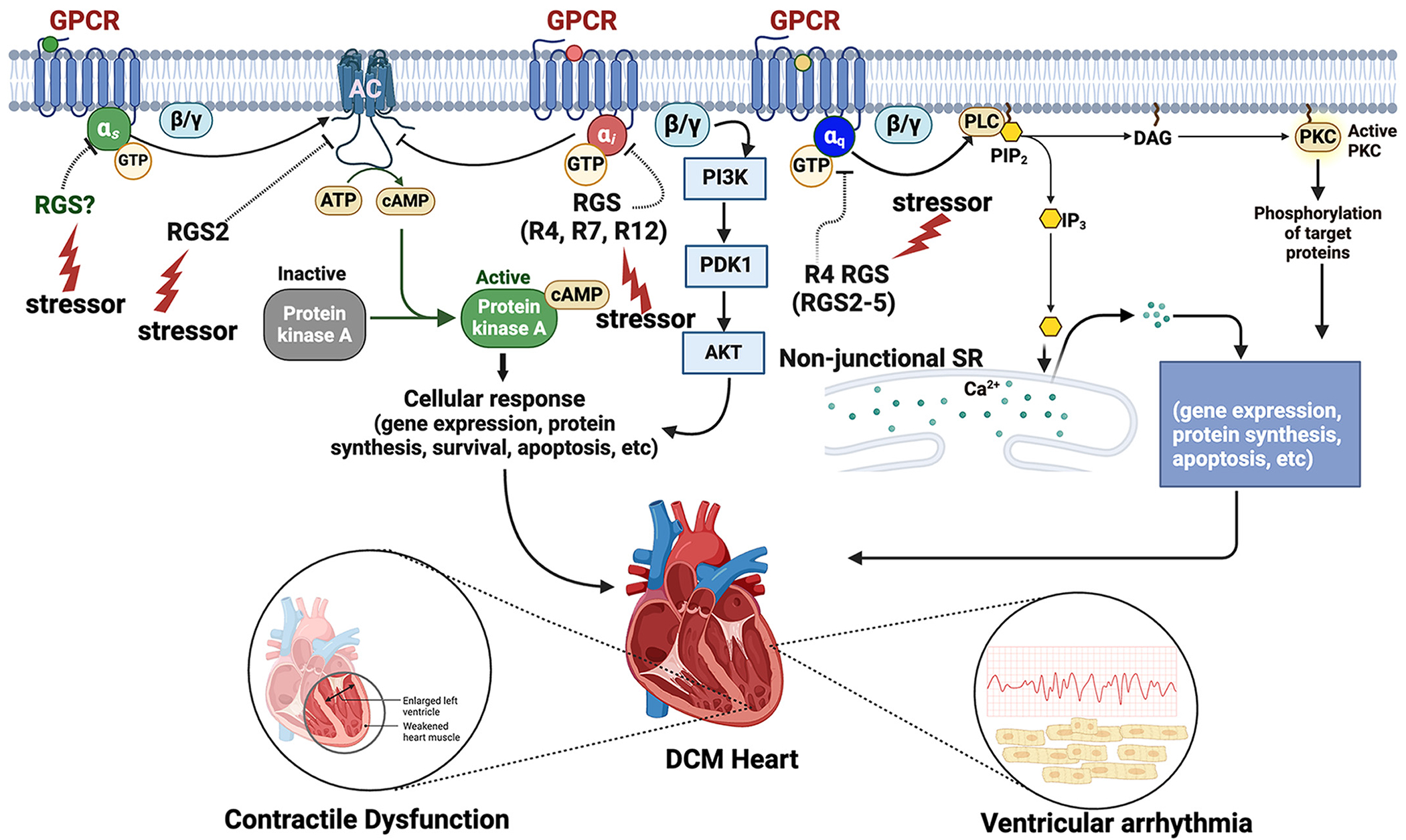
Potential mechanisms by which changes in the expression or function of RGS proteins could lead to defects in signaling pathways that are key to maintaining normal cardiomyocyte structure and function. Studies involving animal models of RGS gene knockout or overexpression have been implicated in the pathogenesis of DCM hallmarks, including left ventricular dilation, cardiomyocyte damage, and ventricular arrhythmia. Thus, exogenous stressors and factors that impinge on the expression or steady-state levels of RGS proteins could contribute to DCM pathogenesis. ai, inhibitory G protein α subunit; α_q_, G_q/11_ class G protein α subunit; α_s_, stimulatory G protein α subunit; βγ, G protein β and γ subunits; AC, adenylyl cyclase; AKT, protein kinase B; Ca^2+^, calcium ion; cAMP, 3′,5′-cyclic adenosine monophosphate; DAG, diacylglycerol; DCM, dilated cardiomyopathy; GPCR, G protein-coupled receptor; stimulatory G protein α subunit; GTP, guanosine triphosphate; IP_3_, inositol 1,4,5-trisphosphate; PDK1, 3-phosphoinositide-dependent kinase 1; PI3K, phosphoinositide 3-kinase; PIP_2_, phosphatidylinositol 4,5-bisphosphate; PKC, protein kinase C; PLC, phospholipase C; RGS, regulator of G protein signaling; Rx, RGS × family; SR, sarcoplasmic reticulum. Figure created with a licensed version of BioRender.com.

As already mentioned, there is growing evidence for the involvement of defects in G protein signaling regulated by both RGS2 and RGS5 in cardiomyopathies; however, studies so far suggest some level of dichotomy in the relative role of these two proteins. On one hand, animal models of pressure-overload cardiac hypertrophy showed that defects in Gα_q/11_ signaling regulation by RGS2 and 5 appears to be key to progression of the maladaptive response to heart failure (45). Conversely, recent studies point to defects in Gα_i/o_ signaling regulation by RGS2 and 5 as a major contributor to DCM pathogenesis (46). Interestingly, RGS2 inhibits Gα_s_-stimulated AC activity, via a non-GAP mechanism but through direct RGS2-AC protein-protein interaction (Fig. 1) (16, 47). Isoproterenol-induced hypertrophy in rat neonatal ventricular cardiomyocytes, via the activation of β-adrenergic receptor (βAR)-Gα_s_ signaling, was accompanied by upregulation of RGS2 mRNA, and RGS2 has been found to have GAP-independent activity in modulating cardiac hypertrophy in this pathway (48). In this setting, isoproterenol-induced activation of downstream ERK1/2 and JNK MAPK pathways leads to compensatory hypertrophy, whereas inhibiting this pathway leads to resistance in developing hypertrophy in mice (48). Phosphorylation of p38 and JNK was found to be protective against hypertrophy in vivo, and that RGS2 promoted this phosphorylation in cardiomyocytes, indicating that the activation of these kinases may be contributing to the antihypertrophic effects of RGS2 in vivo (48). A second GAP-independent mechanism by which RGS2 was shown to mediate antihypertrophic effects was the inhibition of total protein synthesis. This effect was shown to be mediated by a 37-amino acid sequence partly overlapping the RGS domain (RGS2^eb^) that binds and inhibits eukaryotic initiation factor 2B (elF2B). When RGS2^eb^ was expressed in neonatal rat cardiomyocytes, it attenuated protein synthesis and cardiomyocyte hypertrophy induced by agonists of Gα_s_ and Gα_i/o_ coupled GPCRs (23). Conversely, RGS2^eb^ transgenic mice showed resistance to the development of cardiac hypertrophy and a decrease in hypertrophic marker expression (atrial natriuretic peptide and myosin heavy chain-β) along with improved cardiac function (23). Whether such a non-GAP function of RGS2 is involved directly in DCM pathogenesis remains to be further elucidated.

### Genetic Basis of Structural and Functional Remodeling in DCM

Ventricular cardiomyocytes are the primary target of DCM triggers that cause the hallmark structural and functional defects in the myocardium. Some of the most common genetic triggers of DCM are mutations in genes (e.g., *TTN, LMNA*, *MYH7, TNNT2*, and *MYBPC3*) encoding cytoskeletal, sarcomeric, and nuclear envelope proteins such as titin, lamin A/C, myosin heavy chain 7, troponin T2, and myosin binding protein C3 in human cardiomyocytes (2–4). In most cases, the genetic mutations result in truncated protein variants due to insertion/deletion (in/del), frameshift, or nonsense mutations, or splice variants (49). Because some of these proteins serve as scaffolds or confer cytoskeletal elasticity, the generation of truncated variants can lead to protein complex disassembly that underlies the structural and biomechanical defects in DCM. For example, mutations in *LMNA*, the gene encoding lamin A/C, also lead to truncated protein synthesis. *LMNA* encodes two lamin isoforms, A and C, because of alternative splicing (50–52). Lamins are intermediate filament proteins localized at the subnuclear membrane. Lamin proteins selfassemble by forming hetero- or tetra-dimers and stabilize the nuclear internal lamina. Truncated lamins encoded by DCM-associated *LMNA* mutated genes often have defective *α*-helix that precludes hetero- or tetra-dimerization and subsequent protein multimerization for the stabilization of the nuclear internal lamina (51, 52). Mutations in *LMNA* that lead to DCM include the H222P mutation, which was shown to cause increased ERK1/2 phosphorylation (pERK1/2) in mice, and activation of ERK1/2 was, in turn, found to mediate cofilin-1 phosphorylation (53). Cofilins are depolymerizing factors that regulate actin cytoskeleton by promoting the dissociation of actin monomers to increase actin filament turnover. In the H222P mutated *LMNA* mouse model, ERK1/2 activation led to phosphorylation of cofilin-1, activating its F-actin depolymerizing function in cardiomyocytes and in turn leading to the pathogenesis of left ventricular dysfunction in DCM (53). It was then concluded that ERK1/2 signaling activation leads to the development of LMNA cardiomyopathy (53). However, the mechanism of how the mutation leads to increased activation of ERK1/2 is still unclear. Recent studies have reported that some patients with familial DCM harbor mutations in genes encoding titin binding partners, such as myosin and cardiac ankyrin repeat protein 1 (ANKRD-1), which are crucial for recruiting regulatory proteins, including protein kinases (54, 55). However, it is unclear whether such mutations are sufficient to trigger DCM. Nevertheless, such mutations may alter how the central titin scaffold is regulated by posttranslational modification events to affect protein function (49, 56, 57). For instance, titin compliance is dependent, at least partially, on the degree of phosphorylation on multiple protein kinase A (PKA) and/or G (PKG)-mediated phosphorylation sites in the giant protein. Increased PKA/G-mediated phosphorylation can lead to a shift toward a hyperphosphorylated, and less compliant pool of titin isoforms associated with stiff extracellular matrix, as occurs in diastolic dysfunction, as DCM progresses to heart failure.

In addition, roughly 1 in 4 DCM cases (and even higher in adult cases) is a result of mutations in *TTN*, the gene encoding the largest human protein, titin (4 MDa) (49, 58). In striated muscle, including cardiomyocytes, titin forms an elastic polypeptide chain and firmly anchors actin and myosin to the Z-disk. Titin-based stretching of the sarcomere during diastole is postulated to, at least partially, preactivate and prime myocytes for subsequent contraction, thereby serving as a key determinant of contractile force generation in cardiomyocytes (56, 59). Truncation of titin due to gene mutations may disrupt such a critical biomechanical role leading to contractile dysfunction that characterizes DCM. Patients with truncating titin variants (TTNtv) had increased interstitial myocardial fibrosis and nonsustained ventricular tachycardias, and also presented with enhanced cardiac hypertrophy, as well as ventricular arrhythmias (60). The primary kinases for titin, PKA and PKG, are downstream effectors of signaling via heterotrimeric G proteins, thus suggesting a potential mechanism for the regulation of functional titin by proteins that regulate or finetune G protein signaling. Consistent with this postulation, a recent study of mice dually lacking RGS2 and RGS5 (*Rgs2/5* dbKO) reported a set of phenotypes that are hallmarks of DCM, including left ventricular (LV) dilatation and contractile dysfunction, accompanied by higher rates of excitation-contraction coupling abnormalities in LV cardiomyocytes, with increased arrhythmia burden and sudden death in *Rgs2/5* dbKO mice (46). In addition to these structural and functional changes in cardiomyocytes from *Rgs2/5* *dbKO* mice, forskolin-stimulated cAMP generation in *Rgs2/5 dbKO* LV cardiomyocytes was also blunted and was associated with increased activity of inhibitory G proteins (46). A more recent study from the same group reported what appeared to be microscopic evidence of a higher rate of titin disorganization in LV cardiomyocytes from single *Rgs2* KO and *Rgs2/5 dbKO* mice (61). However, it remains to be determined whether the actions of RGS proteins, directly or indirectly, have any role in the regulation of titin and/or related complexes, or whether aberrations in the expression and/or function of RGS proteins, particularly RGS2 and 5, or other related RGS proteins in the same family, play a causal role in the pathogenesis of DCM via their regulatory effects in the organization or function of molecular complexes involving titin in LV cardiomyocytes.

### Genetic Mutations Associated with Ventricular Arrhythmias in DCM

Besides single mutations in *TTN, LMNA*, and other genes found in several DCM cases, there are reports of mutational “hotspots” in titin and sarcomere protein binding partners that are implicated in the disease etiology. Mutations in the actin-binding protein, filamin C, and RNA-binding motif protein 20 (RBM20) have been identified in 1%–5% of all DCM cases with a severe phenotype and often associated with ventricular arrhythmias and sudden cardiac death (5, 55, 59, 62, 63). For example, mutations in the *SCN5A* gene encoding the cardiac sodium channel 1.5 (Na_v_1.5), have been associated with triggered activity during repolarization, when the mutation is a gain-of-function (64). D1275N, a loss-of-function mutation in *SCN5A*, was found to be associated with DCM originally in a large family affected by the condition and various arrhythmias and even led to the development of cardiomyopathy at 12 wk after birth in mice (64). Also, certain mutations in the regulatory protein phospholamban (PLN) have also been identified and implicated in familial DCM, with a high degree of mutation penetrance and disease severity (5, 65–67). Nonetheless, the implication of PLN mutational hotspots in DCM is particularly interesting, given its role in regulating Ca^2+^ uptake via sarco/endoplasmic reticulum Ca^2+^-ATPase 2a (SERCA2a), thereby affecting cytoplasmic Ca^2+^ handling (68). As is well documented, PLN is posttranslationally modified by PKA-mediated phosphorylation, which turns off the inhibitory effect of phospholamban toward SERCA to promote Ca^2+^ uptake into the sarcoplasmic reticulum (SR) and facilitate excitation-contraction coupling (68–71). Cardiomyocyte inotropy and chronotropy are both driven, in large part, by the kinetics and magnitude of SERCA-dependent cytoplasmic Ca^2+^ reuptake by junctional SR to the extent that mutations that alter the regulatory role of phospholamban towards SERCA2a can lead to arrhythmias (71–73). Consistent with this concept, a previous study showed that *PLN* R14 deletion mutation in patients diagnosed with DCM was associated with arrhythmogenic phenotype and sudden cardiac death at a young age (74). Other human PLN mutations have also been identified in patients with DCM, including R25C and R9C (66, 67). *PLN* R25C is a gain-of-function mutation that leads to “super-inhibition” of SERCA2a and diminished affinity of SERCA2a for Ca^2+^ in cardiomyocytes from transgenic rats overexpressing the mutation. Together, these functional effects lead to SR Ca^2+^ leak and increase the susceptibility to stress-induced ventricular arrhythmias in DCM carriers of the mutation (66). In contrast, *PLN* R9C is a missense mutation identified in patients with DCM with refractory congestive heart failure, and transgenic mice carrying *Pln* R9C recapitulate human DCM with sudden cardiac death (67). Mechanistic cellular and biochemical studies using isolated ventricular cardiomyocytes from these mice reported a delayed cytoplasmic Ca^2+^ clearance attributable to trapping of PKA by *Pln* R9C mutant protein, thus blocking PKA-mediated PLN phosphorylation required for SERCA2a disinhibition and activity (67). Interestingly, PLN can be phosphorylated by PKG, albeit at sites distinct from PKA phosphorylation sites (75, 76). However, the diverse functional phenotype described above for *PLN* mutations suggests that PKG-mediated phosphorylation does not have much of a compensatory role at baseline in DCM cardiomyocytes harboring those *PLN* mutations, though such a hypothesis has not been tested in diseased cells carrying those *PLN* mutations. In addition, whether the activity of such an alternative, kinase-dependent regulation of PLN can be elevated by manipulating the activity of upstream signaling components, including G proteins, is worth investigating to gain deeper insight into disease mechanisms and for identifying novel therapeutic targets.

### Nongenetic Alterations Identified in DCM Pathogenesis

Virtually all studies to date indicate that nongenetic causes of primary acquired DCM involve inflammatory destruction of cardiomyocytes mediated by maladaptive immune response to a viral myocardial infection (myocarditis) or sterile chronic inflammation due to autoimmunity (1, 3–5). In either case, T cell activation in the myocardium can directly cause cardiomyocyte damage and subsequent pathological remodeling and a decline in contractile function (7, 77, 78). Anticancer therapies that employ immune checkpoint inhibitors are implicated in idiopathic DCM pathogenesis, as these agents block tumor necrosis factor (TNF)-mediated inhibition of cytotoxic T cells (79). Interestingly, G protein signaling has been shown to be critical to T cell activation, migration, and cytotoxicity, and RGS2 expression increases in T cells upon activation. Mice lacking RGS2 (*Rgs2*^−/−^) have impaired T cell response to viral infection, a phenotype that is underlined by decreased T cell proliferation and production of IL-2 (80). Recent studies also reported that RGS2 coordinates with a long noncoding RNA, HIF-1α inhibitor at translation level (HITT), to stimulate the translation of the ligand (PDL-1) for immune checkpoint receptor, programmed cell death 1 (PD-1). In this role, RGS2 was shown to facilitate T cell-mediated cytotoxicity in a PDL-1-dependent manner (81). Although such a role of RGS2 may be potentially beneficial as a target to enhance cancer immunotherapy, it is conceivable that cardiac-specific homing or recruitment of T cells with increased RGS2 expression or function can predispose to myocardial injury and DCM as occurs in anthracycline-induced cardiomyopathy (82). Thus, the DCM phenotype identified in *Rgs2*^−/−^ and *Rgs2/5 dbKO* mice may not be due to the loss of these RGS proteins in the cardiomyocytes themselves but likely due to aberrant activity of adaptive immune response involving T cells in the myocardium. Moreover, a recent study by Basak et al. (35) found that cardiac RGS7 expression was upregulated in patients with a history of chemotherapy and showed some of the hallmarks of DCM. In a follow-up study investigating the mechanisms behind anthracycline-induced cardiotoxicity, the same group of investigators identified RGS7 as a cardiac inflammatory signaling amplifier, at least partly, by promoting Ca^2+^ overload and the release of inflammatory cytokines such as TNFα and myocardial damage (35, 36). Conversely, knocking down RGS7, either in intact mice or cultured human or murine cardiomyocytes, markedly reduced cardiomyocyte inflammation and oxidative stress (36). The studies mentioned above, thus, provide a solid rationale for further investigations that deepen the understanding of RGS proteins in acquired DCM.

Recreational drug use and chronic alcohol consumption are additional factors other than myocarditis and autoimmunity that are identified as causes of primary acquired DCM. Contractile dysfunction, myocardial fibrosis, and epicardial fat deposition are common structural features of alcohol-induced cardiomyopathy (4, 5, 77). These structural and functional aberrations are reported to be a result of alcohol acting directly on cardiomyocytes to stimulate oxidative stress, edema, and apoptosis, and the adverse effects on the innate immune system (83–88). Methamphetamines and other sympathomimetics that stimulate the sympathetic nervous system can also be cardiotoxic by inducing cardiomyocyte injury leading to DCM (89–91). Sustained activation of the sympathetic nervous system can cause overstimulation of adrenergic receptors (AR) or inefficient regulation of signaling downstream of G_s_- and G_i_/_o_-coupled catecholamine receptors in cardiomyocytes. Along this line, decreased myocardial expression of βAR in failing hearts with DCM has been reported (92, 93). In addition, increased G_i/o_ signaling that suppresses cAMP generation in cardiomyocytes of failing hearts has also been reported (94, 95). Increased Gα_i/o_ activity in DCM cardiomyocytes with downregulated βAR may seem a counterintuitive mechanism for the decline in cAMP generation and signaling. However, G_i_/_o_ signaling can be activated by non-GPCRs, whereas the kinetics and amplitude of Gα_i/o_ activity are modulated by GAPs, including RGS proteins (96, 97). Thus, a change in the balance between the expression and/or activity of Gα_i/o_ selective non-GPCR activators and GAPs in DCM may be an underlying mechanism for the decline in cAMP signaling. Consistent with this idea, recent studies have reported the expression of G_i/o_-selective non-GPCR activators in multiple cells in the myocardium, with effects on fibroblast protein kinase B (AKT) signaling in ischemic heart disease and regulation of ventricular L-type calcium channel (LTCC) activity (98–100). However, the expression, activity, or role of G_i/o_-selective non-GPCR activators in DCM-related heart failure is relatively less explored. In contrast, a comparative investigation of cardiac gene expression of RGS proteins in nonfailing donor hearts and in failing hearts from patients with DCM and ischemic cardiomyopathy identified differential expression of RGS proteins with preferential GAP activity toward G_i/o_, including RGS2–5 of the R4 family and RGS6 of the R7 family (43). The recent report of DCM phenotype in *Rgs2*^−/−^ and *Rgs5*^−/−^ mice raises the question whether environmental factors and lifestyles (including the increasing popularity and use of recreational drugs such as methamphetamine and fentanyl) could potentially impinge on the expression or function of cardiac RGS proteins, thereby increasing the risk of DCM in individuals harboring disease gene variants (Fig. 1).

### Vascular-Specific Alterations in DCM

Vascular abnormalities in DCM are usually seen in acquired, idiopathic DCM, and is characterized by impaired angiogenesis and vasculogenesis of the coronary microvasculature. Historically, vascular abnormalities in DCM are mostly viewed as a consequence but not causative in DCM pathogenesis, given that DCM is generally considered a primary myocardial disease of cardiomyocytes (7, 58). This notion was likely born out of the observation that the common hallmarks of peripheral vascular dysfunction in several forms of heart disease and disorders are distinctly different from abnormal changes in the coronary vasculature associated with poor myocardial perfusion in DCM (101). In addition, intrinsic changes in cardiomyocyte regulatory proteins and transcriptional regulators that impair cardiomyocyte structure have been shown to cause vascular rarefaction and decreased endothelial capillary density in DCM (102). Nonetheless, a close correlation between abnormal myocardial blood flow and heart failure resulting from DCM is well established (58, 103, 104). Furthermore, recent close reexaminations of DCM pathophysiology, focusing on acquired primary DCM, have led to a new hypothesis, whereby genetic or structural changes in various cell types of the vessel wall serve as a DCM disease initiation site.

#### Endothelial function.

Endothelial cells (EC) in the myocardium release several factors, including cytokines, nitric oxide (NO), and other relaxing factors, and trophic factors that are key to the maintenance and regulation of cardiomyocyte structural integrity and contractility. The coronary microcirculation communicates with the myocardium most directly via the juxtaposition of cardiac endothelial capillaries and cardiomyocytes to mediate the transport of multiple factors. Such factors range from oxygen and nutrients on demand, vasoactive agents including endothelin and NO, and trophic factors such as TNF-α and vascular endothelial growth factor (VEGF), all from the circulation, and capillary, endocardial, and endothelial cells for cardiomyocyte growth and repair (58, 105–107). A recent study by Matsa et al. (108) reported the identification of three functional endothelial NO synthase gene (*NOS3*) polymorphisms, T786C in the 5’-untranslated region, 27 bp VNTR in intron 4, and G894T in exon 7 in patients with DCM. These variants were found to be recessive with a sevenfold increased risk for DCM relative to controls, and were also associated with increased NO production, thus increasing the risk of cardiomyocyte damage and hypercontractility due to oxidative/nitrosative stress (108). Interestingly, endothelial NO generation is a Ca^2+^-dependent process and is regulated, at least partly, by GPCR signaling involving G_s_ and G_q/11_ classes of G proteins (109). In addition, previous studies reported endothelial dysfunction involving the loss of G_q/11_ and G_i/o_ regulation by RGS2 in small arteries from *Rgs2*^−/−^ mice (110, 111). Putting in perspective, the increased DCM risk associated with *NOS3* polymorphisms and nitrosative stress could be related to the degree to which mutated *NOS3* gene products are amenable to regulation by NOS activators and modulators, including some of the components of GPCR signaling mentioned above.

#### Vascular smooth muscle.

**Figure 2.**
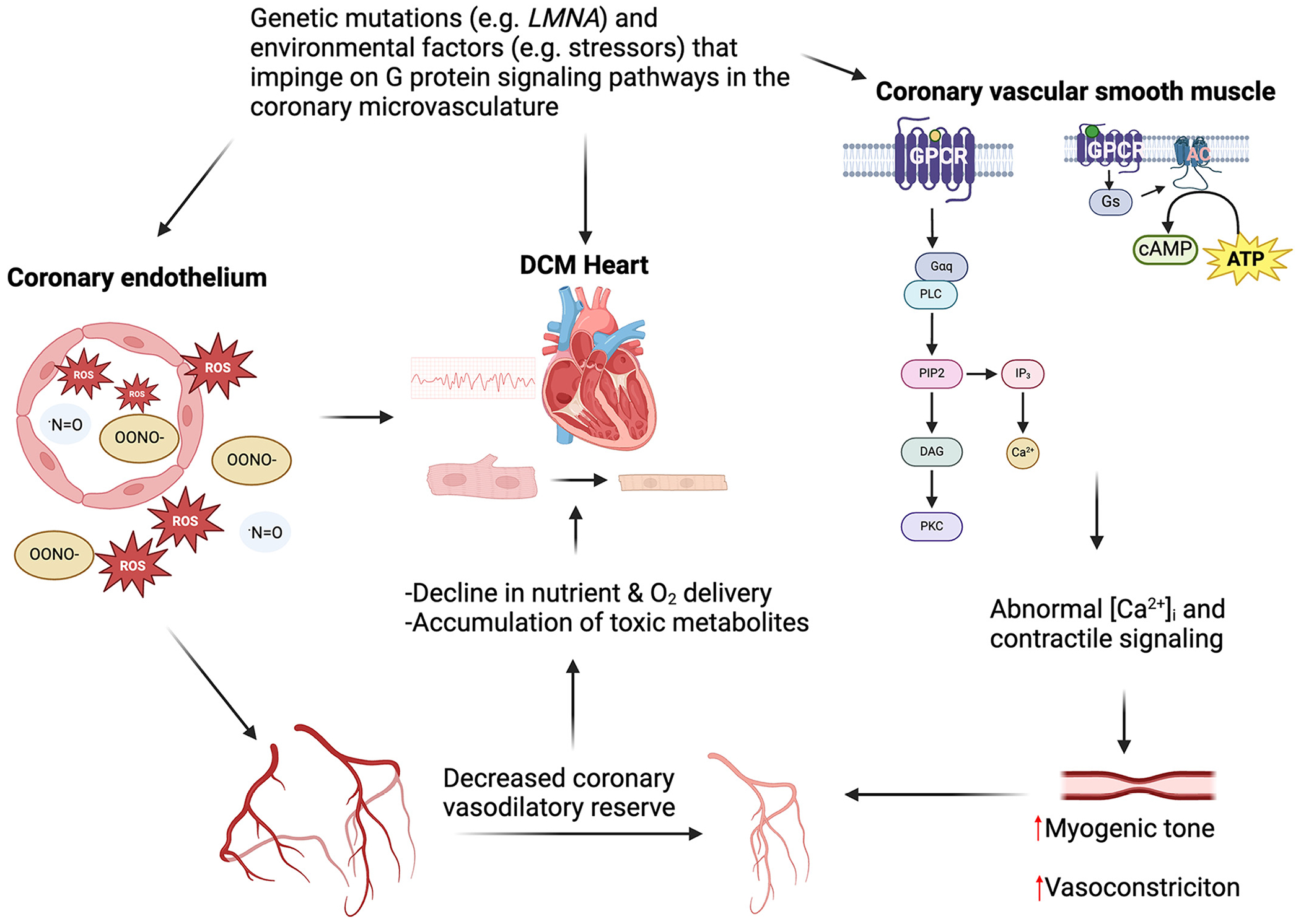
Contribution of vascular dysfunction to DCM pathogenesis. Certain mutations in cardiomyocytes implicated in DCM pathogenesis are also found in cells of the coronary microcirculation. Vascular dysfunction resulting from such mutations and environmental stressors, including chronic alcohol use and certain medications, are associated with decreased coronary vasodilatory reserve and increased oxidative and nitrosative stress and decline in oxygen and nutrient delivery to the myocardium that together can contribute to cardiomyocyte damage and dysfunction. α_s_, stimulatory G protein α subunit; AC, adenylyl cyclase; ATP, adenosine triphosphate; cAMP, 3′,5′-cyclic adenosine monophosphate; Ca^2+^, calcium ion; DAG, diacylglycerol; DCM, dilated cardiomyopathy; Gα_q_, G_q/11_ class G protein α subunit; GPCR, G protein-coupled receptor; IP_3_, inositol 1,4,5-trisphosphate; *LMNA*, human laminin A/C gene; NO, nitric oxide; O_2_, molecular oxygen; ONOO^−^, peroxynitrite; PIP_2_, phosphatidylinositol 4,5-bisphosphate; PKC, protein kinase C; PLC, phospholipase C; ROS, reactive oxygen species. Figure created with a licensed version of BioRender.com.

The vascular smooth muscle layer of arteries in the coronary microcirculation responds to a variety of stimuli including endogenous vasoactive ligands and stretch, thereby setting coronary vascular tone and vessel lumen diameter to facilitate the perfusion of intramyocardial capillaries juxtaposed to cardiomyocytes. Nitric oxide released from the vascular endothelium promotes vasodilatation and increased coronary blood flow by activating vascular smooth muscle intrinsic vasodilatory signaling involving the stimulation of soluble guanylyl cyclase for the generation of cGMP that in turn leads to PKG-dependent phosphorylation of downstream effector proteins (112–115). Most studies of the cardiac microvascular functional changes in DCM to date have focused on the vasodilatory reserve of the coronary circulation. The assessment of coronary blood flow and coronary artery vasodilatory response upon stimulation of muscarinic GPCRs of the endothelium with acetylcholine, pharmacologically induced hyperemia with dipyridamole, or the stimulation of vascular smooth muscle adenosine receptors have reported decreased vasodilatory reserve resulting from decreased NO bioavailability due to endothelial dysfunction, and also due partly to impaired vascular smooth muscle relaxation mechanisms (8, 116). Interestingly, a few of the genetic mutations implicated in cardiomyocyte abnormalities in DCM are also found in other muscle types, including vascular smooth muscle cells, and are also associated with coronary vascular dysfunction and decreased coronary vasodilatory reserve in patients with DCM, suggesting a contributory role for the vascular defects (8, 116). Cardiomyopathy leading to heart failure in patients with Duchenne (DMD) and Becker (BMD) muscular dystrophies is an example of DCM resulting from mutations that affect cardiomyocytes and other muscle cell types including vascular smooth muscle cells (8, 117, 118). Both DMD and BMD are muscle wasting diseases caused by mutations in the gene encoding dystrophin, a sarcolemmal protein connecting myocyte contractile machinery to the extracellular matrix via a complex of dystrophin-associated glycoproteins, which are a family of integral membrane proteins also known as sarcoglycans (117, 119). Studies using animal models of DMD have reported abnormal arterial remodeling leading to impaired contractile function and exaggerated vasodilatory response to exogenous NO (120, 121). A mechanistic study in the mdx mouse model of DMD reported enhanced activity of the vasodilatory BK channel, mediated by increased ryanodine receptor type 2 (RYR2)-mediated SR Ca^2+^ release (121). These molecular changes were reported to underlie the impairment of spontaneous myogenic tone and may also account for the hypo-contractile response and exaggerated vasodilatation observed in a prior study (8, 122). These studies demonstrate a close association between the impairment of both the coronary and peripheral vasculature and DCM. Potentially, mutations that lead to the impairment of upstream signaling regulating SR Ca^2+^ release, or dysregulated G_q/11_ and G_i/o_ signaling by RGS proteins, could add to or affect coronary perfusion, predisposing to DCM (46). A recent study of the effects of vascular smooth muscle-specific ablation of *Lmna* in mice provided the strongest evidence yet, supporting vascular dysfunction as a primary trigger of DCM (50). This study by Monte-Monge et al. (50) showed that smooth muscle 22α (SM22α)-mediated *Lmna* deletion led to the recapitulation of DCM disease hallmarks, including ventricular systolic dysfunction, cardiac fibrosis, and premature death. In addition, vessels from *Lmna*^−/−^ mice exhibited impaired contractile and vasodilatory responses reminiscent of vascular dysfunction in patients with DCM and other animal models (8, 116). Intuitively, and based on several studies to date, impaired coronary microvascular function could lead to ischemic stress and myocardial injury due to failure of a compensatory increase in coronary perfusion in response to increased cardiac workload (Fig. 2). However, it remains to be determined how smooth muscle-restricted mutations directly trigger cardiomyocyte injury and/or death leading to ventricular dilation as occurs in DCM.

#### Cardiac fibroblasts and DCM.

Histological analyses of endomyocardial biopsies from heart failure patients have established myocardial fibrosis as a pathological hallmark of DCM. Both interstitial and perivascular fibrosis have been observed in DCM myocardium (123, 124). Myocardial interstitial fibrosis in DCM is a reparative response, whereby dead or damaged cardiomyocytes are replaced by activated myofibroblasts. Myocardial fibrotic lesion in DCM is described as patchy and is associated with degenerated cardiomyocytes and thin-walled, dilated left ventricle (123–125). Perivascular fibrosis, usually more prominent in the intramyocardial coronary arteries, is also found in histopathologic assessments of endomyocardial biopsies from patients and animal models of DCM (58). However, in contrast to interstitial fibrosis, perivascular fibrosis of the intramyocardial arteries is a reactive response triggered by ischemia and metabolic abnormalities in the coronary circulation (123). To date, there is no evidence to suggest that myocardial fibrosis by itself is a primary trigger of DCM. Nonetheless, several reports, including those already cited herein, indicate that fibrosis accelerates DCM progression to heart failure with reduced ejection fraction. Fibrogenesis alters the quality and ratio of collagen fiber types, thereby affecting collagen fiber alignment with ventricular cardiomyocytes that may blunt cardiomyocyte force transmission and manifest as decreased contractile function characteristic of DCM (123, 126). Fibrogenesis in DCM and other forms of cardiomyopathies begins with the activation, proliferation, and transformation of fibroblasts to myofibroblasts (123). Differentiated myofibroblasts synthesize and secrete extacellular matrix (ECM) proteins, mainly procollagen types I and III, into the myocardial interstitium and the perivascular space around intramyocardial vessels, where the secreted propeptides are further processed and crosslinked to mature collagen fibers in the fibrotic lesion (123, 124). Signal transduction events central to cardiac fibrosis are those downstream of the transforming growth factor β1 receptor (TGF-β1R), also known as activin receptor-like kinase (ALK5) (123, 127, 128). Canonically, activated TGF-β1R induces cell proliferation and survival, and epithelial-mesenchymal cell transition involving transcription activation by a series of phosphorylated SMAD proteins (123, 127). Noncanonical TGF-β1R activity triggers signaling downstream of PI3K-AKT-mTOR, Ras-Raf-MAPK, and TGF-β-activated kinase 1 (TAK1)-MAPK/JNK/p38 to induce protein synthesis including ECM proteins, as well as activating cell proliferation and survival, and epithelial-mesenchymal cell transition (123, 127). Signaling via heterotrimeric G proteins and small, single Ras domain-containing g proteins plays a major role in mediating fibroblast activation by diverse stimuli including stretch, paracrine and autocrine factors, and effector molecules of various endocrine systems including angiotensin II (Ang II) from the renin-angiotensin system (RAS), cytokines, chemokines, growth factors, and catecholamines from the sympathetic nervous system (129, 130). Chief among the receptors for these signal molecules is angiotensin receptor type 1 (ATR1). In addition to Ang II from the circulation and from local RAS activation, mechanical stimuli such as produced by pressure-overload myocardial stretch can activate AT1R on myocytes (131, 132). Stretch-activated AT1R induces cardiac fibrosis partly by stimulating the production of the cognate TGF-β1R agonist, TGF-β1, and other profibrotic agonists from fibroblasts that in turn act in an autocrine mechanism (128, 133, 134). Activated AT1R can also induce cardiac fibrogenesis in a ligand-free, stretch-mediated receptor stimulation that transactivates TGF-β1R (128, 133, 134). AT1R-dependent TGF-β1 production is mediated by the activation of G_q/11_ signaling in activated fibroblasts (127, 128). Other agonists that activate G_q/11_ signaling, including endothelin-1 (ET-1), have also been shown to be profibrotic by activating similar pathways as Ang II and stretch in cardiac fibroblasts (127, 128). Interestingly, a previous study by Zhang et al. (22) reported that enhanced regulation of G_q/11_ signaling by RGS2 in cardiac fibroblasts blunted Ang II-induced fibrotic response, including the suppression of cell proliferation and total collagen synthesis in an in vitro model of cardiac fibroblast activation. This suggests that the loss of endogenous regulators of G_q/11_ class G proteins, including RGS2, 4, and 5 of the R4 RGS family that are prominently expressed in the myocardium, could increase the propensity to develop cardiac fibrosis. In line with this hypothesis, other previous studies using *Rgs2*^−/−^ and *Rgs5*^−/−^ mice have reported increased levels of fibrotic lesions after pressure-overload cardiac hypertrophy (28, 30, 135). Whether G_q/11_ signaling is also a key mediator of cardiac fibrosis in DCM is yet to be fully explored. However, it is noteworthy that simulation of sympathetic hyperactivity via systemic infusion of the non-selective βAR agonist, isoproterenol, in mice dually lacking RGS2 and RGS5 led to exacerbated myocardial interstitial fibrosis (136). Cardiac βAR receptors couple to G_s_ and G_i/o_ class G proteins, both of which impinge on cAMP signaling in the myocardium to elicit effects on cardiac structure and function. Regarding fibrosis, previous reports indicate that, in normal physiology, exchange protein activated by cAMP (Epac)1/2 and PKA downstream of G_s_-cAMP signaling, act as negative regulators of fibrosis by activating CREB to inhibit key transcriptional activity for myofibroblast differentiation (129, 137, 138). A previous study from the Osei-Owusu laboratory demonstrated that the loss of RGS2 alone or both RGS2 and RGS5 led to increased G_i/o_ activity and decreased cAMP production in ventricular cardiomyocytes (46). Thus, it is conceivable that dysregulation of G_i/o_ signaling due to the absence of RGS2, RGS5, or both RGS proteins could also lead to the loss of homeostatic negative regulation or profibrotic CREB activation, predisposing to the disinhibition of sympathetic-mediated myocardial fibrosis in DCM. This shows the importance of understanding how the regulation of RGS proteins is related to DCM and other cardiac diseases.

### Current Gaps in the Understanding of the Role of Signal Transduction (G Protein and Non-G Protein) in DCM Etiology and Future Perspectives

G protein signaling regulation is well-established to play crucial roles in maintaining normal physiology. Several studies also implicate abnormal changes in the function of G protein regulatory proteins, including RGS proteins, in the development and progression of multiple diseases. Studies of the biological relevance of RGS proteins since their discovery in the 1990s continue to unveil such disease mechanisms involving RGS proteins. However, the role of RGS proteins in the pathogenesis of DCM, resulting from genetic and nongenetic primary triggers in cardiomyocytes and noncardiac cells of the cardiovascular system, is poorly understood and understudied. Translational studies that delve more deeply into the molecular mechanisms by which changes in G protein regulation are involved in cardiac development and function are key to understanding the etiology of DCM and other cardiomyopathies. The prominent expression of RGS proteins, particularly those of the R4 family highly expressed in the vascular and immune systems, should be exploited in future studies designed to understand noncardiomyocyte origin of DCM. Along these lines, many environmental factors deemed as primary triggers of DCM have the potential or have already been shown to affect the expression and function of R4 RGS proteins in the vascular and immune systems. Therefore, future studies of the pathogenesis of cardiomyopathies should be expanded by utilizing multifaceted approaches geared toward the elucidation of the cellular and molecular mechanisms underlying both G protein and non-G protein signaling regulation by RGS proteins in the myocardium and vascular system of both the coronary and extra-coronary vessels. Such multipronged strategies may unveil novel mechanisms and signaling pathways that could serve as novel therapeutic targets.

## Figures and Tables

**Table 1.** RGS proteins implicated in cardiomyopathies

RGS	RGS Family	G Protein Family	Role in Cardiac Disease	Biological Model	Refs.
RGS1	R4	G_q/11_, G_i/o_	CHD (CVD, CAD)	Patients with cardiac disease; human aortic smooth muscle cells	(18–20)
RGS2	R4	G_q/11_, G_i/o_	Cardiac arrhythmia (atrial and ventricular); cardiac hypertrophy	Arrhythmic rats; murine VCM; transgenic mice (constitutively active Gα_q_^Q209L^); *Rgs2*^−/−^ mice	(21–24)
RGS3	R4	G_q/11_, G_i/o_	Cardiac hypertrophy, heart failure	Neonatal rat cardiomyocytes; SHHF rats	(25)
RGS4	R4	G_q/11_, G_i/o_	Cardiac arrhythmia (atrial); cardiac hypertrophy	*Rgs4*^−/−^ mouse cardiomyocytes; *Rgs4* overexpression in neonatal rat cardiomyocytes	(25–27)
RGS5	R4	G_q/11_, G_i/o_	Cardiac dysfunction and fibrosis; cardiac arrhythmia (ventricular); cardiac hypertrophy	*Rgs5*^−/−^ mice, whole-body and cardiacspecific transgenic *Rgs5* mice; *Rgs5* silencing/KD in mice	(28–30)
RGS6	R7	G_i/o_	Stress-induced cardiomyocyte death; cardiac arrhythmia (sinoatrial); cardiac hypertrophy; cardiac development	*Rgs6* KD/overexpression in mice, primary ventricular mouse cardiomyocytes; *Rgs6*^−/−^ mice, human heart tissue; mouse embryonic fibroblasts	(31–34)
RGS7	R7	G_i/o_	Fibrosis in murine myocardium; left ventricular dysfunction and fibrosis	Overexpression in iPSC-derived cardiomyocytes; KD in AC-16 cells; murine VCM	(35, 36)
RGS10	R12	G_i/o_	Cardiac arrhythmia (atrial)	Rat atrial myocytes	(37)
RGS12	R12	G_i/o_	Cardiac hypertrophy	Human tissue, neonatal rat cardiomyocytes, *Rgs12*^−/−^ mice, *Rgs12* transgenic mice	(38)

CAD, coronary artery disease; CHD, coronary atherosclerotic heart disease; CVD, cardiovascular disease; iPSC, induced pluripotent stem cells; KD, knock-down; RGS, regulator of G protein signaling; SHHF, spontaneously hypertensive heart failure; VCM, ventricular cardiomyocytes.
